# *x*- and *y*-type thioredoxins maintain redox homeostasis on photosystem I acceptor side under fluctuating light

**DOI:** 10.1093/plphys/kiad466

**Published:** 2023-08-22

**Authors:** Yuki Okegawa, Nozomi Sato, Rino Nakakura, Ryota Murai, Wataru Sakamoto, Ken Motohashi

**Affiliations:** Institute of Plant Science and Resources, Okayama University, Kurashiki 710-0046, Japan; Faculty of Life Sciences, Kyoto Sangyo University, Kyoto 603-8047, Japan; Center for Plant Sciences, Kyoto Sangyo University, Kyoto 603-8047, Japan; Faculty of Life Sciences, Kyoto Sangyo University, Kyoto 603-8047, Japan; Faculty of Life Sciences, Kyoto Sangyo University, Kyoto 603-8047, Japan; Institute of Plant Science and Resources, Okayama University, Kurashiki 710-0046, Japan; Faculty of Life Sciences, Kyoto Sangyo University, Kyoto 603-8047, Japan; Center for Plant Sciences, Kyoto Sangyo University, Kyoto 603-8047, Japan

## Abstract

Plants cope with sudden increases in light intensity through various photoprotective mechanisms. Redox regulation by thioredoxin (Trx) systems also contributes to this process. Whereas the functions of *f*- and *m*-type Trxs in response to such fluctuating light conditions have been extensively investigated, those of *x*- and *y*-type Trxs are largely unknown. Here, we analyzed the *trx x* single, *trx y1 trx y2* double, and *trx x trx y1 trx y2* triple mutants in *Arabidopsis* (*Arabidopsis thaliana*). A detailed analysis of photosynthesis revealed changes in photosystem I (PSI) parameters under low light in *trx x* and *trx x trx y1 trx y2*. The electron acceptor side of PSI was more reduced in these mutants than in the wild type. This mutant phenotype was more pronounced under fluctuating light conditions. During both low- and high-light phases, the PSI acceptor side was largely limited in *trx x* and *trx x trx y1 trx y2*. After fluctuating light treatment, we observed more severe PSI photoinhibition in *trx x* and *trx x trx y1 trx y2* than in the wild type. Furthermore, when grown under fluctuating light conditions, *trx x* and *trx x trx y1 trx y2* plants showed impaired growth and decreased level of PSI subunits. These results suggest that Trx *x* and Trx *y* prevent redox imbalance on the PSI acceptor side, which is required to protect PSI from photoinhibition, especially under fluctuating light. We also propose that Trx *x* and Trx *y* contribute to maintaining the redox balance even under constant low-light conditions to prepare for sudden increases in light intensity.

## Teaser

*x*- and *y*-type thioredoxins contribute to preventing the redox imbalance of the photosystem I acceptor side and protecting photosystem I from photoinhibition under fluctuating light.

## Introduction

Plants are often exposed to fluctuating light. When the light intensity exceeds the plant's photosynthetic capacity, the excess energy results in the formation of reactive oxygen species (ROS), ultimately causing photoinhibition. Chloroplasts have various adaptive mechanisms to avoid photoinhibition and optimize their photosynthetic performance (Tikhonov 2015).

In the light reactions of photosynthesis, electrons generated by the splitting of water at photosystem II (PSII) are transferred to NADP^+^ through the cytochrome *b*_6_*f* complex and PSI, resulting in the production of NADPH. Electron transport is coupled with the translocation of protons, generating a proton gradient across the thylakoid membrane (ΔpH), which is utilized in ATP synthesis and is also required for the induction of photoprotective mechanisms (Tikhonov 2015). Acidification of the thylakoid lumen slows down electron transport through the cytochrome *b*_6_*f* complex, preventing overreduction of the primary electron donor of PSI (P700), in a mechanism called photosynthetic control (Stiehl and Witt 1969; Foyer et al. 2012). Thylakoid lumen acidification also induces thermal dissipation of the excess light energy absorbed by PSII antennae, in a mechanism that prevents oxidative stress to PSII (Muller et al. 2001; Li et al. 2002). This process is monitored as the nonphotochemical quenching (NPQ) of chlorophyll fluorescence (Niyogi et al. 1998). PSI cyclic electron transport (PSI-CET) is required to induce both these mechanisms and protect PSI from photoinhibition (Chaux et al. 2015; Shikanai and Yamamoto 2017). The *Arabidopsis* (*Arabidopsis thaliana*) *proton gradient regulation 5* (*pgr5*) mutant, which is defective in the major pathway of PSI-CET, cannot induce photosynthetic control and thermal dissipation and is sensitive to high and fluctuating light conditions (Munekage et al. 2004; Suorsa et al. 2012; Yamamoto and Shikanai 2019).

Redox regulation by thioredoxin (Trx) systems also contributes to the flexible modulation of photosynthesis in response to rapid changes in light intensity (Cejudo et al. 2021; Gonzalez et al. 2021). In chloroplasts, the two Trx systems function in a coordinated manner (Nikkanen and Rintamaki 2019; Cejudo et al. 2021; Yoshida et al. 2022). The NADPH-Trx reductase C (NTRC) pathway is unique to chloroplasts and predominantly reduces 2-Cys peroxiredoxin (2-Cys Prx) using NADPH as an electron donor (Serrato et al. 2004; Perez-Ruiz et al. 2006). Subsequently, 2-Cys Prx scavenges hydrogen peroxide and converts it into water. A mutant defective in NTRC (*ntrc*) exhibits growth retardation even under constant low-light conditions owing to the redox imbalance in chloroplasts (Perez-Ruiz et al. 2017).

In the second Trx system, namely, the ferredoxin (Fd)/Trx pathway, Trxs are reduced by photosynthetically reduced Fd via Fd-Trx reductase (FTR), and the reduced Trxs then reduce target enzymes, thereby modulating enzyme structure and function (Schurmann and Buchanan 2008). The *Arabidopsis* chloroplast contains five different types of Trx: two Trx *f* (*f*1 and *f*2), four Trx *m* (*m*1, *m*2, *m*3, and *m*4), Trx *x*, two Trx *y* (*y*1 and *y*2), and Trx *z*. Most photosynthesis-related enzymes, including those in the Calvin–Benson–Bassham (CBB) cycle, are active during the day and inactive at night. Numerous in vivo and in vitro studies have demonstrated that Trx *f* and Trx *m* mainly modulate the activity of these enzymes (Buchanan 2016; Geigenberger et al. 2017; Nikkanen and Rintamaki 2019). Rapid activation of CBB cycle enzymes by Trx *f* is needed to induce photosynthesis efficiently during dark-to-light transitions (Naranjo et al. 2016). We have previously reported that Trx *f* and the PGR5-dependent PSI-CET pathway function cooperatively in this process and prevent photoinhibition (Okegawa et al. 2020). Alternatively, Trx *m*1 and *m*2 are required for full photosynthetic activity during the high-light phases of fluctuating light (Thormahlen et al. 2017).

In contrast, little is known about the roles of Trx *x* and Trx *y* under fluctuating light conditions. In *Arabidopsis* stroma, Trx *f* and Trx *m* account for more than 90% of the total chloroplast Trx proteins, whereas Trx *x* and Trx *y* represent only 6.3% and 1.3% of the total, respectively (Okegawa and Motohashi 2015). Trx *x* and Trx *y* are functionally related and mainly serve as reducing substrates for antioxidant enzymes (Collin et al. 2003, 2004; Geigenberger et al. 2017). Trx *y*2 functions as an electron donor for Prx Q, methionine sulfoxide reductase, and NADPH-dependent monodehydroascorbate reductase (Collin et al. 2004; Laugier et al. 2013; Vanacker et al. 2018). The Trx *y*–deficient mutant (*trx y1 trx y2*) showed increased sensitivity to high light and drought. An in vitro experiment revealed Trx *x* to be the most efficient reductant of 2-Cys Prx (Collin et al. 2003), whereas NTRC seemed to be primarily responsible for the reduction of 2-Cys Prx in vivo (Pulido et al. 2010). In contrast to the *ntrc* mutant, the Trx *x*–deficient mutant (*trx x*) did not show any visible growth defects (Pulido et al. 2010). However, introducing the *trx x* mutation into the *ntrc* mutant background exacerbated the *ntrc* growth defects even under normal growth conditions (Ojeda et al. 2017). Loss of Trx *y* also slightly exacerbated the *ntrc* growth defects (Jurado-Flores et al. 2020). These results suggest that Trx *x* and Trx *y* also have important functions in photosynthesis.

Here, we characterized the *trx x* single, *trx y1 trx y2* double, and *trx x trx y1 trx y2* triple mutants. In *trx x*, the acceptor-side limitation of PSI, Y(NA), was increased by fluctuating light treatment, resulting in PSI photoinhibition. This phenotype was more prominent in *trx x trx y1 trx y2*. When grown under fluctuating light conditions, *trx x* and *trx x trx y1 trx y2* exhibited severe growth defects. Our results suggest that Trx *x* and Trx *y* contribute to the PSI acceptor-side regulation.

## Results

### Lack of Trx *x* and Trx *y* did not affect plant growth under constant light conditions

**Figure 1.**
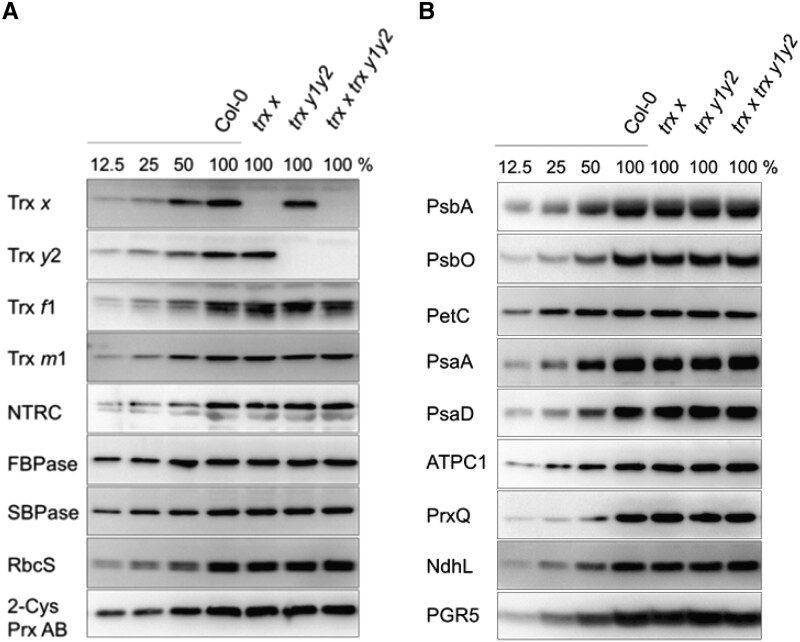
Immunoblot analysis of photosynthesis-related proteins in WT Col-0, *trx x*, *trx y1y2*, and *trx x trx y1y2* plants. Chloroplasts were fractionated into stromal fractions and thylakoid membranes. Ten micrograms of stromal protein **(A)** or thylakoid membrane proteins corresponding to 1.0-*µ*g chlorophyll **(B)** were loaded per lane, as well as a dilution series of WT proteins. Antibodies used are indicated on the left.

To investigate the physiological functions of Trx *x* and Trx *y* in vivo, we generated a mutant lacking both Trx *x* and Trx *y*. First, we isolated a *trx x* mutant (GK_179A03) containing a transfer-DNA (T-DNA) insertion at the 30-bp upstream region of the *Trx x* translation initiation codon (Supplemental Fig. S1). Meanwhile, we crossed the *trx y1* and *trx y2* T-DNA single mutants (Laugier et al. 2013) to generate a *trx y1 trx y2* double mutant (hereafter referred as *trx y1y2*). We then crossed *trx x* with *trx y1y2* to obtain the *trx x trx y1 trx y2* triple mutant (hereafter referred as *trx x trx y1y2*). In the triple mutant, we did not detect any Trx *x* or Trx *y*2 protein by immunoblot analysis (Fig. 1A). The accumulation of Trx *y*1 in the wild-type (WT) chloroplasts was below the limit of immunoblot detection (Okegawa and Motohashi 2015); therefore, we were unable to confirm loss of this protein in *trx x trx y1y2*. However, the *trx y1* mutant used in this study was reported to have complete loss of *Trx y1* expression, as determined by reverse transcription (RT)-PCR (Laugier et al. 2013). We grew all three mutants under long-day conditions at a light intensity of 50 to 60 *µ*mol photons m^−2^ s^−1^ for 23 days. All mutants exhibited similar growth as the WT in terms of fresh weight (Table 1). Similarly, there was no difference in fresh weight between WT and mutant plants under short-day or continuous-light conditions (Table 1). Consistent with these results, the levels of photosynthesis-related proteins were comparable among all the genotypes (Fig. 1).

**Table 1.** Growth of WT Col-0, *trx x*, *trx y1y2*, and *trx x trx y1y2* mutant plants under long-day, short-day, and continuous-light conditions

	Fresh weight (mg)		
	Long day	Short day	Continuous light
Col-0	48.7 ± 6.5^a^	42.1 ± 7.5^b^	45.6 ± 4.8^c^
*trx x*	46.3 ± 5.6^a^	43.5 ± 7.0^b^	47.2 ± 5.1^c^
*trx y1y2*	48.3 ± 7.2^a^	42.5 ± 9.0^b^	44.0 ± 6.6^c^
*trx x trx y1y2*	48.3 ± 8.1^a^	45.5 ± 8.6^b^	44.6 ± 3.8^c^

Seedlings were grown under long-day conditions (16-h light/8-h dark) for 23 d, short-day conditions (8-h light/16-h dark) for 36 d or continuous-light conditions for 19 d. The light intensity was 50 to 60 *µ*mol photons m^−2^ s^−1^ under all conditions. Each value is the mean ± Sd of 10 independent plants. Means with the same letters are not significantly different between genotypes according to Tukey's test, *P* < 0.05.

### Electron transport was mildly affected in *trx x* and *trx x trx y1y2* during steady-state photosynthesis

**Figure 2.**
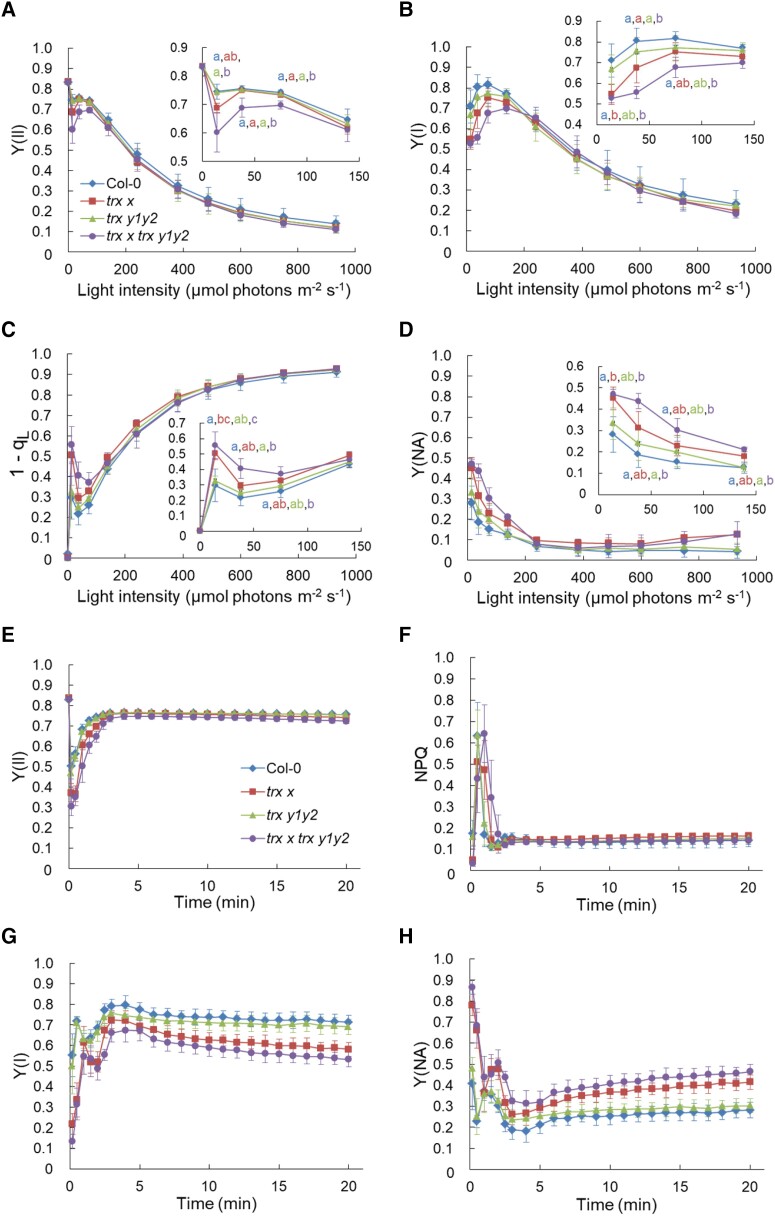
Effect of Trx *x* and Trx *y* deficiencies on steady-state photosynthesis. Chlorophyll fluorescence and P700 parameters in WT Col-0, *trx x*, *trx y1y2*, and *trx x trx y1y2* plants were measured. **A to D**) Light intensity dependence of PSII and PSI photosynthetic parameters. **A)** Effective quantum yield of PSII [Y(II)]. **B)** Photochemical quantum yield of PSI [Y(I)]. **C)** Reduction level of the PQ pool (1 − *q_L_*). **D)** Acceptor-side limitation of PSI [Y(NA)]. Results for low light intensities are shown in close-up in the insert. Each value is the mean ± Sd of 3 independent plants. Different letters indicate statistical differences confirmed by the Tukey–Kramer test (*P* < 0.05). **E to H)** Y(II), non-photochemical quenching (NPQ) of chlorophyll fluorescence, Y(I), and Y(NA), respectively, were measured upon illumination at 64 *µ*mol photons m^−2^ s^−1^ for 20 min. Each data point represents the mean ± Sd (*n* = 6 to 8 independent plants).

Subsequently, we measured chlorophyll fluorescence and absorption changes in P700 using a Dual-PAM-100 system to investigate the effects of Trx *x* and Trx *y* deficiencies on steady-state photosynthesis. The maximum quantum yield of PSII (*F_v_*/*F_m_*), which is an indicator of functional PSII, did not differ among the four genotypes (WT, 0.834 ± 0.005; *trx x*, 0.835 ± 0.007; *trx y1y2*, 0.836 ± 0.010; and *trx x trx y1y2*, 0.834 ± 0.009; each value is the mean ± Sd of 10 independent plants). Light intensity dependence of PSII and PSI photochemistry was analyzed (Fig. 2). At light intensities below 100 *µ*mol photons m^−2^ s^−1^, the effective quantum yield of PSII [Y(II)], estimated from chlorophyll fluorescence, was not affected in *trx x* and *trx y1y2* but was lower in *trx x trx y1y2* than in the WT (Fig. 2A). Similarly, the Y(I) parameter of P700 absorbance changes, which is often used to estimate the effective quantum yield of PSI, was also affected in *trx x trx y1y2* under low-light conditions (Fig. 2B). Unlike Y(II), Y(I) was lower in *trx x* than in the WT but only at a light intensity of 14 *µ*mol photons m^−2^ s^−1^. The 1 − *q_L_* parameter, which is used to estimate the reduction level of the plastoquinone (PQ) pool (Kramer et al. 2004), was higher in *trx x trx y1y2* than in the WT at light intensities below 100 *µ*mol photons m^−2^ s^−1^ (Fig. 2C). The Y(NA) parameter represents the acceptor-side limitation of PSI, and its value is determined by the balance between electron inflow into and outflow from PSI (Klughammer and Schreiber 1994). *trx x* and *trx x trx y1y2* had higher Y(NA) values than the WT under low-light conditions (Fig. 2D), indicating that the acceptor side of PSI was moderately reduced compared to the WT. In contrast, there were no differences in NPQ or donor-side limitation of PSI [Y(ND)] under low-light conditions between the WT and mutants (Supplemental Fig. S2, A and B). Meanwhile, under higher light intensities, we observed no differences in any of these photosynthetic parameters among the four genotypes. Y(NA) was slightly higher in *trx x* and *trx x trx y1y2* than in the WT above 700 *µ*mol photons m^−2^ s^−1^, but the difference was not statistically significant (Fig. 2D).

In the above analysis (Fig. 2, A to D), we increased the intensity of actinic light (AL) in a step-wise manner every 2 min, so the decrease in Y(I) and Y(II) in *trx x trx y1y2* under low light might have been caused by a delay in the induction of photosynthesis. Thus, we assessed Y(I) and Y(II) during the induction of photosynthesis at a light intensity of 64 *µ*mol photons m^−2^ s^−1^, which is almost the same as the plant growth light intensity (Fig. 2, E to H; Supplemental Fig. S2, C and D). After the shift from dark to light, the increase in Y(II) was delayed in *trx x trx y1y2* (Fig. 2E). Within 1 min after the onset of AL, *trx x* also showed lower Y(II) than the WT. However, *trx x* and *trx x trx y1y2* reached the WT level of Y(II) within a few minutes, indicating a slight delay in photosynthetic induction. Correspondingly, the relaxation of NPQ was slightly delayed in *trx x* and *trx x trx y1y2* (Fig. 2F). In contrast, Y(I) remained consistently lower in *trx x* and *trx x trx y1y2* than in the WT and did not reach the WT level even 20 min after the onset of AL (Fig. 2G). Similarly, Y(NA) was slightly higher in *trx x* and *trx x trx y1y2* than in the WT (Fig. 2H). These results indicated that lack of Trx *x* and Trx *y* caused the acceptor-side limitation of PSI under steady-state photosynthesis.

### Photoactivation of FBPase and SBPase was slightly delayed in *trx x trx y1y2* during the induction of photosynthesis

**Figure 3.**
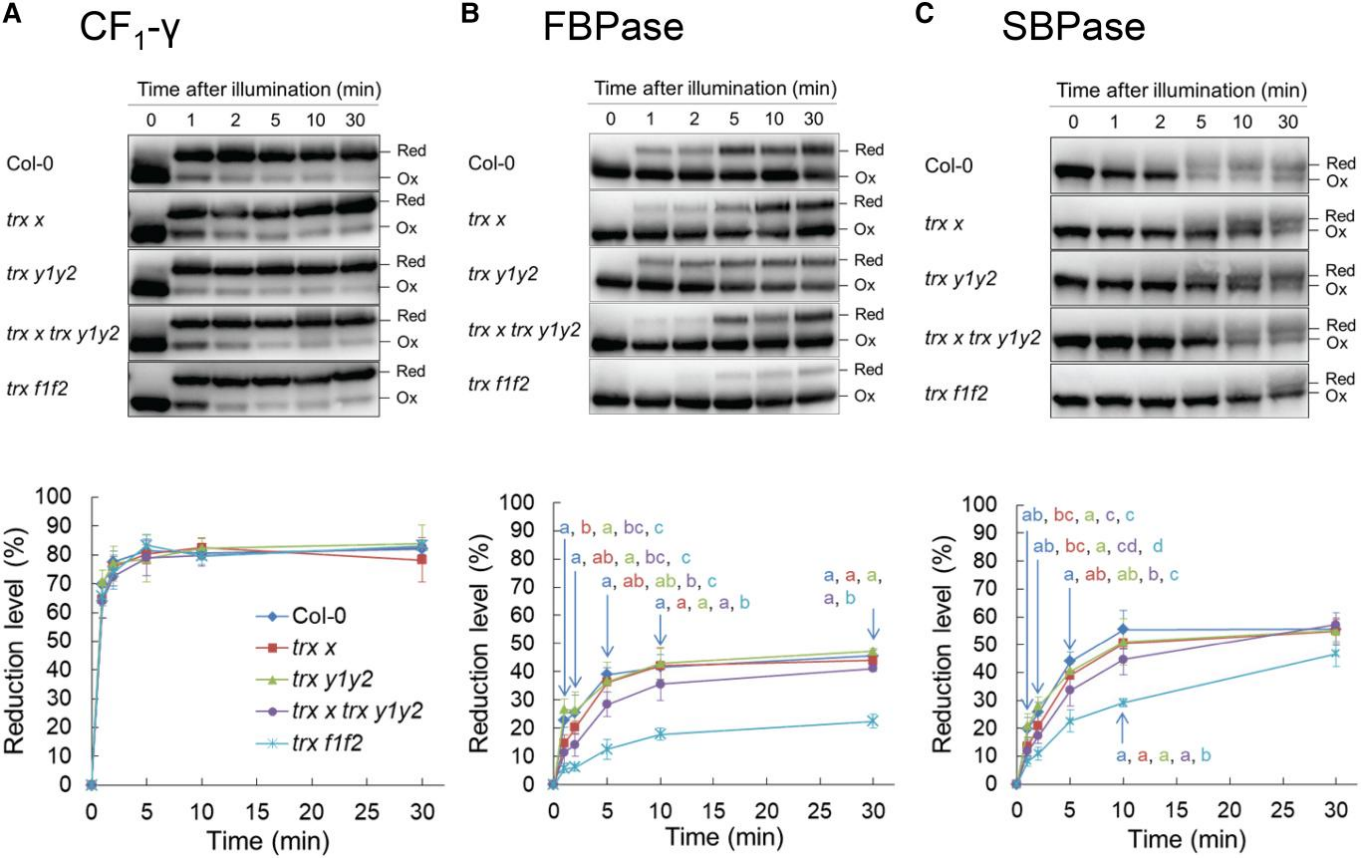
Effect of Trx *x* and Trx *y* deficiencies on activation of CBB cycle enzymes during photosynthesis induction. Light-dependent reduction of thiol enzymes in WT Col-0, *trx x*, *trx y1y2*, *trx x trx y1y2*, and *trx f1f2* plants was examined. The redox states of the ATP synthase subunit CF_1_-γ **(A)**, FBPase **(B)**, and SBPase **(C)** were detected by immunoblot analysis. The reduction levels of thiol enzymes are indicated as a percentage of the total protein that was reduced. Each value represents the mean ± Sd (*n* = 3 to 5 independent plants). Different letters indicate statistical differences confirmed by the Tukey–Kramer test (*P* < 0.05). Ox, oxidized; Red, reduced.

The Trx *f*–deficient mutant (*trx f1trx f2*; hereafter referred as *trx f1f2*) showed a delayed induction of photosynthesis owing to suppressed activation of CBB cycle enzymes (Naranjo et al. 2016; Okegawa et al. 2020; Sekiguchi et al. 2020). To investigate whether the lack of Trx *x* and Trx *y* affects the light-dependent activation of these enzymes, we examined the photoreduction of several thiol enzymes during photosynthesis induction (Fig. 3). We used *trx f1f2* as a control. The CF_1_-γ subunit of chloroplast ATP synthase requires reduction by Trxs for enzyme activation (Nalin and Mccarty 1984). In all genotypes, CF_1_-γ was rapidly reduced upon illumination and reached a steady-state reduction level within 5 min (Fig. 3A). There was no difference in the final reduction levels between the WT and mutants. Consistent with previous studies (Okegawa et al. 2020), the reduction of fructose 1,6-bisphosphatase (FBPase) and sedoheptulose-bisphosphatase (SBPase) was considerably suppressed in *trx f1f2* (Fig. 3, B and C). Similarly, the reduction levels of FBPase and SBPase were lower in *trx x trx y1y2* than in the WT during the first few minutes after the onset of illumination (Fig. 3, B and C). However, their final reduction levels were almost identical to those of the WT. These results suggest that Trx *x* and Trx *y* also contribute to the rapid activation of CBB cycle enzymes during the induction of photosynthesis.

### The PSI acceptor side was greatly limited in *trx x* and *trx x trx y1y2* under fluctuating light conditions

**Figure 4.**
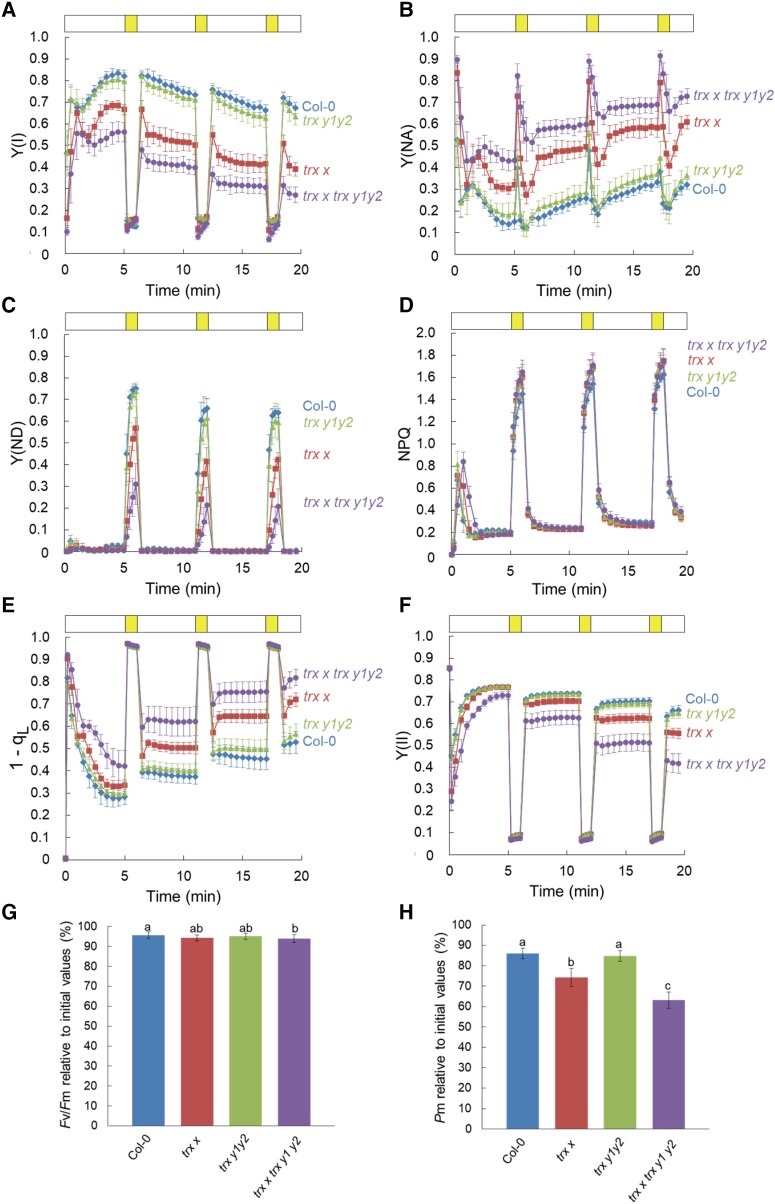
Impact of fluctuating light exposure on photosynthesis in WT Col-0, *trx x*, *trx y1y2*, and *trx x trx y1y2* plants. PSI and PSII parameters under fluctuating light were measured. Four-week-old plants grown under long-day conditions were exposed to cycles of low light (white bar, 54 *µ*mol photons m^−2^ s^−1^) and high light (yellow bar, 1,455 *µ*mol photons m^−2^ s^−1^). **A) to F)** represent Y(I), Y(NA), donor-side limitation of PSI [Y(ND)], NPQ, 1 − *q_L_*, and Y(II), respectively. Each data point represents the mean ± Sd (*n* = 6 to 11 independent plants). **G, H)** PSII and PSI photoinhibition. The *F_v_*/*F_m_* and *P_m_* values were compared before and after the fluctuating light treatment. Each value is the mean *±*Sd (*n* = 12 to 18 independent plants). Different letters indicate statistical differences confirmed by the Tukey–Kramer test (*P* < 0.05).

Loss of Trx *x* and Trx *y* mildly affected PSI parameters under steady-state photosynthesis (Fig. 2). To examine the effect of *trx x* and *trx y1y2* mutations on PSI photoprotection, we analyzed the photosynthetic parameters under fluctuating light conditions known to cause more pronounced photoinhibition of PSI than PSII (Yamamoto et al. 2016). Specifically, we exposed the plants to periodic fluctuating light consisting of 5 min of low light (54 *µ*mol photons m^−2^ s^−1^) and 1 min of high light (1,455 *µ*mol photons m^−2^ s^−1^) using a DUAL-PAM system (Fig. 4). Consistent with the result of time-course analysis (Fig. 2G), Y(I) was lower in *trx x* and *trx x trx y1y2* than in the WT already during the first low-light phase (Fig. 4A). In the WT, Y(I) gradually decreased throughout the fluctuating light experiment, and the *trx y1y2* mutant showed a similar trend. However, Y(I) decreased more dramatically in *trx x* and *trx x trx y1y2* (Fig. 4A). After 3 cycles of fluctuating light, the values of Y(I) dropped to about 60% of that in the WT in *trx x* and to about 40% in *trx x trx y1y2* (WT, 0.67 ± 0.02; *trx x*, 0.39 ± 0.03; *trx y1y2*, 0.63 ± 0.04; *trx x trx y1y2*, 0.27 ± 0.04; each value is the mean ± Sd of 6 to 11 independent plants). These results suggest that PSI was more sensitive to fluctuating light in *trx x* and *trx x trx y1y2*. During fluctuating light, Y(NA) was always higher in *trx x* and *trx x trx y1y2* than in the WT (Fig. 4B). The acceptor side of PSI was more severely limited in *trx x trx y1y2* compared to *trx x*. After the shift from the first low-light phase to the high-light phase, Y(NA) was drastically increased in all genotypes (Fig. 4B), indicative of reduction of P700 by enhanced electron flow toward PSI. However, Y(NA) of the WT was the same as or lower than that of *trx x trx y1y2* at the low-light phase and quickly decreased to the level before high-light exposure, probably because of induction of photosynthetic control and NPQ (Fig. 4, C and D). Y(ND) is often used to estimate the operation of photosynthetic control (Yamamoto and Shikanai 2019). In *trx x trx y1y2*, Y(NA) did not decrease below the level before high-light exposure in the first and second high-light phases and increased further in the subsequent low-light phase (Fig. 4B). This is consistent with the fact that induction of Y(ND) was strongly suppressed in *trx x trx y1y2* (Fig. 4C). With increasing Y(NA), 1 − *q_L_* was much higher in *trx x* and *trx x trx y1y2* compared to the WT (Fig. 4E), and Y(II) was noticeably lower (Fig. 4F).

After the fluctuating light treatment, we evaluated photoinhibition of PSII and PSI by measuring *F_v_*/*F_m_* and *P_m_* (the level of maximum oxidizable P700), respectively (Fig. 4, G and H). The *F_v_*/*F_m_* values were above 93% of those before the fluctuating light treatment in all genotypes (Fig. 4G). PSI showed more photoinhibition than PSII from fluctuating light. Even in the WT, *P_m_* decreased to 85% of the initial levels (Fig. 4H). *trx y1y2* had identical values to the WT (85%), whereas PSI was more severely photoinhibited in *trx x* and *trx x trx y1y2*. The values of *P_m_* decreased to 75% and 64% in *trx x* and *trx x trx y1y2*, respectively (Fig. 4H). These results suggest that Trx *x* and Trx *y* protect PSI from photoinhibition by alleviating the acceptor-side limitation of PSI under fluctuating light.

To investigate this possibility, we crossed the *proton gradient regulation 1* (*pgr1*) mutant with *trx x trx y1y2* and obtained a *pgr1 trx x trx y1y2* quadruple mutant (Supplemental Fig. S3). The *pgr1* mutant has an amino acid alteration in the Rieske subunit of the cytochrome *b*_6_*f* complex and a decreased electron transport rate due to its hypersensitivity to low luminal pH (Munekage et al. 2001; Jahns et al. 2002). As a result, P700 is more oxidized in *pgr1*. In *pgr1 trx x trx y1y2*, Y(I) was considerably higher than in *trx x trx y1y2* in the low-light phases and almost the same as in the WT (Supplemental Fig. S3A). The acceptor-side limitation of PSI was also largely alleviated in *pgr1 trx x trx y1y2*, as indicated by a lower Y(NA) than in *trx x trx y1y2* (Supplemental Fig. S3B). The strong induction of Y(ND) by the *pgr1* mutation, especially in the high-light phase, probably suppressed electron transport to PSI in *pgr1 trx x trx y1y2* (Supplemental Fig. S3C). In parallel with the recovery of Y(I), Y(II) and 1 − *q_L_* were also restored (Supplemental Fig. S3, D and E). As a result, PSI photoinhibition was significantly suppressed in *pgr1 trx x trx y1y2* (Supplemental Fig. S3G). These results strongly suggest that Trx *x* and Trx *y* prevent the overreduction of the PSI acceptor side and protect PSI from photoinhibition.

### *The trx x* and *trx x trx y1y2* mutants showed severe growth defects under fluctuating light

**Figure 5.**
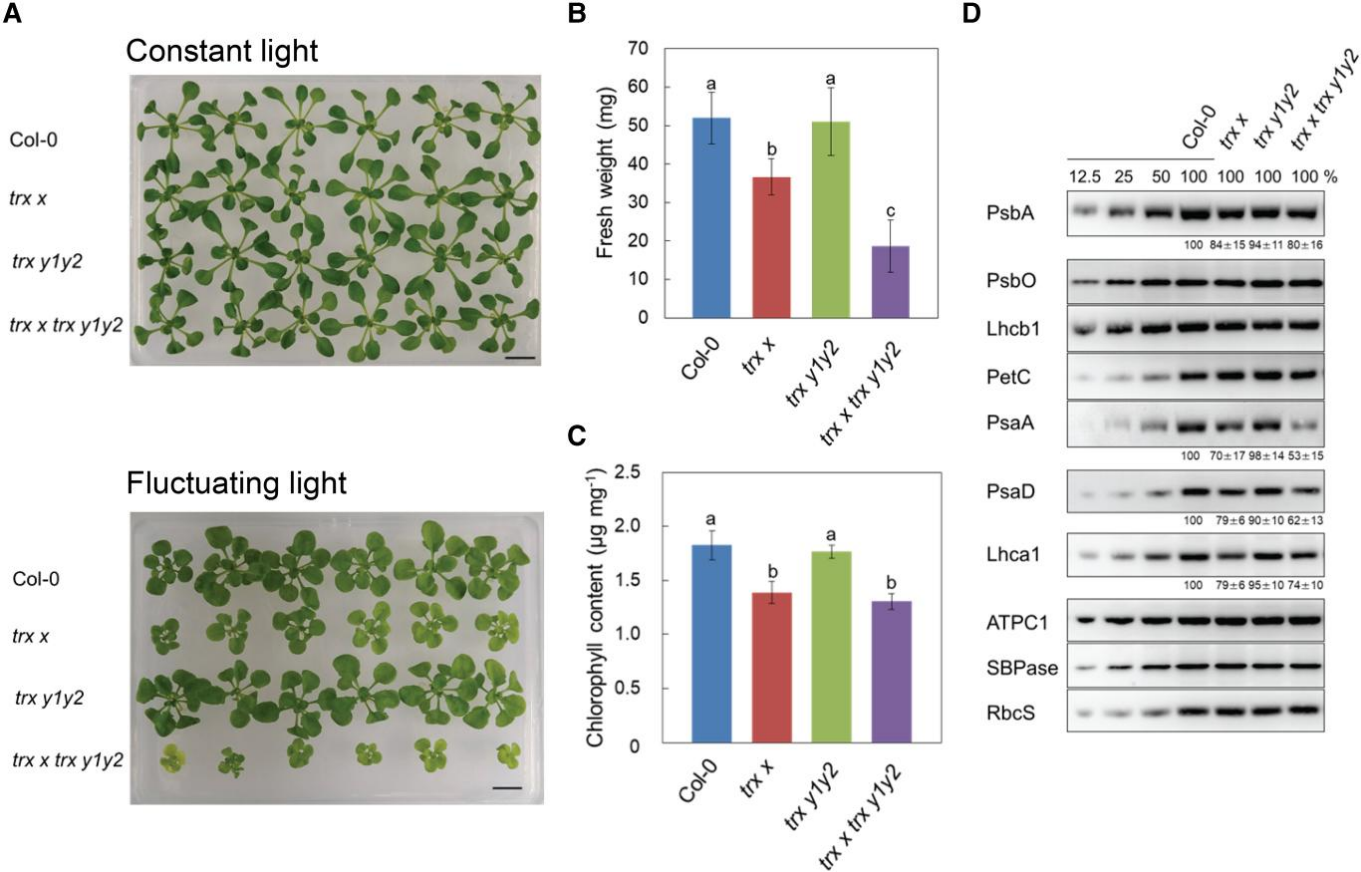
Visible phenotypes of WT Col-0, *trx x*, *trx y1y2*, and *trx x trx y1y2* plants under fluctuating light conditions. **A)** Seedlings were grown on MS medium for 19 d in growth chambers under continuous light (50 to 60 *µ*mol photons m^−2^ s^−1^) or fluctuating light cycles of 5 min of low light (30 *µ*mol photons m^−2^ s^−1^) and 1 min of high light (500 *µ*mol photons m^−2^ s^−1^). Scale bars: 10 mm. **B)** Fresh weight of seedlings grown under fluctuating light. Each value is shown as the mean ± Sd (*n* = 11 to 13 independent plants). **C)** Chlorophyll content of seedlings, per unit fresh weight, grown under fluctuating light. Each value is the mean ± Sd of 3 independent samples. Different letters indicate statistical differences confirmed by the Tukey–Kramer test (*P* < 0.05). **D)** Immunoblot analysis of photosynthesis-related proteins in plants grown under fluctuating light. The same amount of total leaf protein (10 *µ*g) was loaded per lane, as well as a dilution series of WT proteins. Experiments were performed using at least 3 independent sample preparations, and representative results are shown. Shown are the averages ± Sd of the intensity of each band relative to WT Col-0 (100%). Values are given only for those proteins that showed statistically significant differences from the WT (Tukey–Kramer test, *P* < 0.05).

Next, we grew the WT, *trx x*, *trx y1y2*, and *trx x trx y1y2* plants under artificial fluctuating light to investigate the effect of fluctuating light on plant growth (Fig. 5). Indeed, the mutant plants were indistinguishable from WT plants under constant light conditions, whereas the growth of *trx x* and especially *trx x trx y1y2* was severely affected under fluctuating light (Fig. 5A). The fresh weight was decreased to 73% and 36% of that in the WT in *trx x* and *trx x trx y1y2*, respectively (Fig. 5B). Furthermore, the chlorophyll content in *trx x* and *trx x trx y1y2* leaves was only 76% and 71% of that in WT leaves, respectively (Fig. 5C).

We also examined PSI protein accumulation in plants grown under fluctuating light. *trx y1y2* accumulated the same levels of all proteins assayed as the WT (Fig. 5D). There also were no distinct differences between the WT, *trx x*, and *trx x trx y1y2* in the amounts of PetC (a subunit of the cytochrome *b*_6_*f* complex), ATPC1 (a γ subunit of the ATP synthase), SBPase, or RbcS (Rubisco small subunit). Among the PSII complex proteins, the amounts of PsbA in *trx x* and *trx x trx y1y2* were decreased to about 84% and 80% of that in the WT, respectively, while the levels of PsbO and Lhcb1 did not differ from those in the WT. In contrast, the amounts of PSI complex subunits were considerably decreased in *trx x* and *trx x trx y1y2* (Fig. 5D). The levels of PsaA in *trx x* and *trx x trx y1y2* were approximately 70% and 53%, respectively, of that in the WT, while those of PsaD were 79% and 62%, respectively, of the WT levels. Furthermore, the amounts of the light-harvesting complex protein Lhca1 in *trx x* and *trx x trx y1y2* were decreased by about 79% and 74%, respectively, from that in the WT. These results suggest that the PSI photoinhibition caused by fluctuating light significantly affects plant growth in *trx x* and *trx x trx y1y2* mutants, as indicated by our analysis of photosynthetic parameters (Fig. 4).

### In vivo redox status of 2-Cys Prx was unaffected in *trx x* and *trx x trx y1y2*

**Figure 6.**
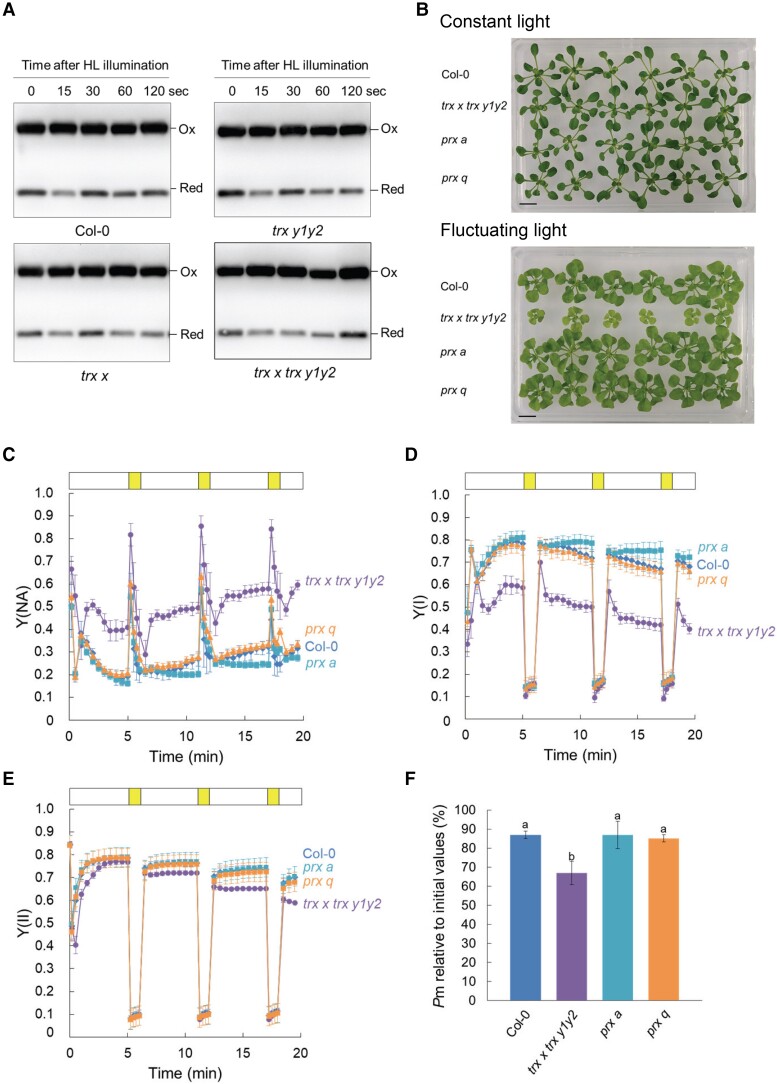
In vivo redox state of 2-Cys Prx in in WT Col-0, *trx x*, *trx y1y2*, and *trx x trx y1y2* plants. **A)** Seedlings were exposed to low light (30 *µ*mol photons m^−2^ s^−1^) for 30 min and then to high light (500 *µ*mol photons m^−2^ s^−1^) for 120 s and collected at the indicated time points. Reduced thiols were blocked by *N*-ethylmaleimide. The redox state of 2-Cys Prx was detected by immunoblot analysis. HL, high light; Ox, oxidized; Red, reduced. **B)** Visible phenotypes of WT Col-0, *trx x trx y1y2*, *prx a*, and *prx q* plants. Seedlings were grown on MS medium in growth chambers under continuous light for 19 d or fluctuating light for 21 d. Scale bars: 10 mm. **C to F)** Analysis of PSI and PSII parameters under fluctuating light in WT Col-0, *trx x trx y1y2*, *prx a*, and *prx q* plants grown under long-day conditions. **C) to E)** represent Y(NA), Y(I), and Y(II), respectively. Each data point represents the mean ± Sd (*n* = 5 to 10 independent plants). **F)** PSI photoinhibition. Each value is the mean *±*Sd (*n* = 6 to 8 independent plants). Different letters indicate statistical differences confirmed by the Tukey–Kramer test (*P* < 0.05).

How then do Trx *x* and Trx *y* protect PSI from photoinhibition? In the chloroplast redox network, 2-Cys Prx is abundant and serves as an electron sink (Dietz 2011; Perez-Ruiz et al. 2017). Although NTRC mainly contributes to the reduction of 2-Cys Prx in vivo (Pulido et al. 2010; Supplemental Fig. S4A), Trx *x* and Trx *y* may be the major electron donors for 2-Cys Prx under specific conditions, such as fluctuating light. To evaluate this possibility, we determined the redox status of 2-Cys Prx during the transition from low to high light in vivo, which simulated the same change in light intensity as when grown under fluctuating light. The 2-Cys Prx monomer corresponds to the reduced form and the dimer to the fully or partially oxidized form. At a low light intensity of 30 *µ*mol photons m^−2^ s^−1^, the ratio of the reduced form was approximately 20% in all genotypes (Fig. 6A; Supplemental Fig. S4B). An increase in light intensity to 500 *µ*mol photons m^−2^ s^−1^ did not significantly change the reduction level of 2-Cys Prx, although a slight increase or decrease was observed (Fig. 6A). There were no differences in the reduction levels of 2-Cys Prx with changes in light intensity between the WT and mutants (Fig. 6A; Supplemental Fig. S4B). These results indicated that, unlike the loss of NTRC, loss of Trx *x* and Trx *y* did not significantly impact the reduction of 2-Cys Prx to be detectable by immunoblot analysis.

To further investigate the relationships between Trx *x* and Trx *y* and 2-Cys Prx, a knockout mutant of 2-Cys Prx A (*prx a*) was grown under fluctuating light. *Arabidopsis* has two almost identical types of 2-Cys Prx, named A and B. Mutant plants with decreased amount of total 2-Cys Prx to 4% of the WT showed growth defects even under normal growth conditions (Perez-Ruiz et al. 2017), whereas the *prx a* mutant still accumulated about 20% of 2-Cys Prx due to 2-Cys Prx B accumulation and grew as well as WT under constant light (Fig. 6B; Supplemental Fig. S4C). Since Prx Q is known to be reduced primarily by Trx *y* and also by Trx *x* in vitro (Collin et al. 2004), Prx Q–deficient mutant (*prx q*) was also analyzed (Fig. 6B; Supplemental Fig. S4D). In contrast to *trx x trx y1y2*, both *prx a* and *prx q* showed similar growth with WT when grown under fluctuating light conditions (Fig. 6B; Supplemental Fig. S4E). We also measured the PSI and PSII photosynthetic parameters of *prx a* and *prx q* under fluctuating light (Fig. 6, C to F; Supplemental Fig. S4, F to I). Unlike *trx x trx y1y2*, *prx a* and *prx q* did not show the acceptor-side limitation of PSI or lower Y(I) and Y(II) than the WT (Fig. 6, C to E). Rather, Y(NA) was slightly lower in *prx a* than in the WT in the second and third low-light phases (Figs. 6C). As well, PSI photoinhibition was not observed in *prx a* and *prx q* (Fig. 6F). These results suggest that an insufficient supply of reducing equivalents to 2-Cys Prx or Prx Q is not the major cause of growth inhibition in *trx x* and *trx x trx y1y2*.

### Activity of NDH complex–dependent PSI-CEF was unaltered in *trx x* and *trx x trx y1y2*

**Figure 7.**
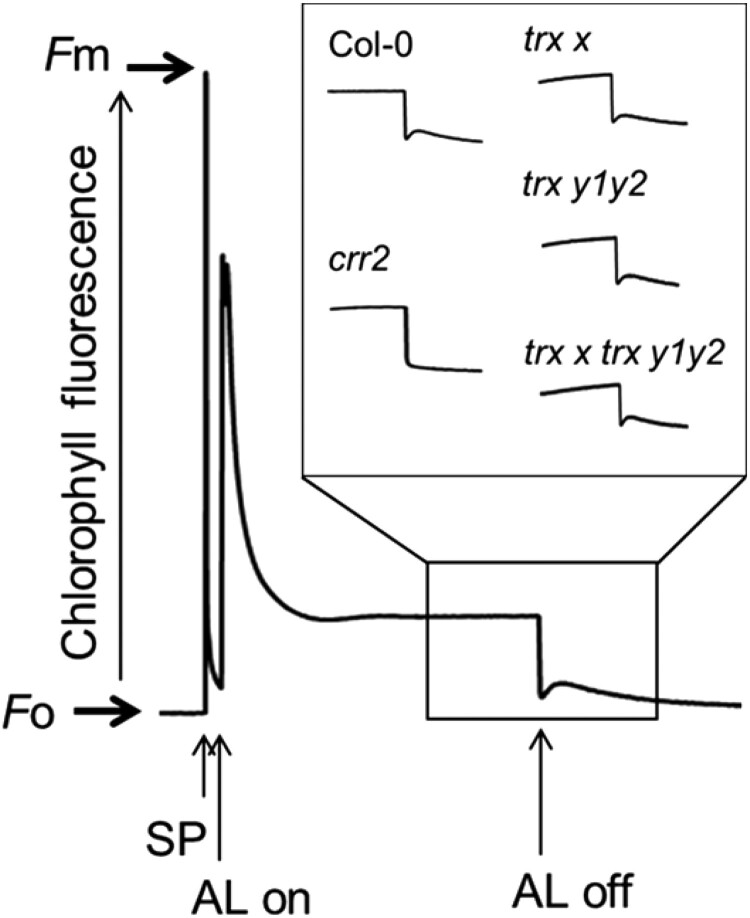
The transient increase in chlorophyll fluorescence dependent on NDH activity in WT Col-0, *crr2*, *trx x*, *trx y1y2*, and *trx x trx y1y2* plants. Leaves were exposed to AL (50 *µ*mol photons m^−2^ s^−1^) for 5 min. After illumination, the subsequent transient increase in fluorescence caused by NDH activity was monitored. A typical trace of chlorophyll fluorescence in WT Col-0 is shown. Insets are magnified traces from the boxed area. The fluorescence levels were normalized with respect to the *F_m_* level. SP, saturating pulse.

Recently, the PSI-CET pathway dependent on the NADH dehydrogenase-like (NDH) complex has been reported to contribute to the protection of PSI under fluctuating light (Kono and Terashima 2016; Yamori et al. 2016; Zhou et al. 2023). The PSI parameters of the *chlororespiratory reduction 2* (*crr2*) mutant, which is defective in the NDH-dependent pathway, under fluctuating light were similar to those of *trx x* and *trx x trx y1y2*, which motivated us to test whether Trx *x* and Trx *y* are involved in modulating the activity of the NDH complex. NDH activity can be monitored as a transient increase in chlorophyll fluorescence by PQ reduction after AL illumination (Shikanai et al. 1998). This is because the NDH complex retains the ability to donate electrons to the PQ pool in the dark. In *crr2* plants, the post-illumination increase in chlorophyll fluorescence was absent (Fig. 7). In contrast, we observed an increase in fluorescence in the WT, *trx x*, *trx y1y2*, and *trx x trx y1y2* plants (Fig. 7). Furthermore, *crr2* did not show any growth defects under fluctuating light (Supplemental Fig. S5A); there was no difference in the fresh weights between WT and *crr2* plants (Supplemental Fig. S5B). These results suggest that loss of Trx *x* and Trx *y* does not affect the activity of the NDH complex, at least under our experimental conditions.

## Discussion

The *trx x* and *trx y1y2* mutants exhibit WT phenotypes under constant light conditions (Jurado-Flores et al. 2020). Consequently, a detailed analysis of steady-state photosynthesis is lacking for *trx x* and *trx y1y2*. Simultaneous analysis of the PSI and PSII photosynthetic parameters revealed that the acceptor side of PSI was slightly reduced under low-light conditions in *trx x*, but not in *trx y1y2* (Fig. 2). This phenotype was more evident under fluctuating light (Fig. 4). Furthermore, the introduction of the *trx y1y2* mutation into the *trx x* mutant background more severely damaged PSI (Fig. 4). These results indicate that the *trx x* mutation has a greater impact on photosynthesis than the *trx y1y2* mutation, suggesting that Trx *y* may have a compensatory role against Trx *x* at least under the examined conditions; however, it is unclear whether Trx *x* and Trx *y* are involved in the same processes. Alternatively, a longer high-light phase of fluctuating light might reveal phenotypic differences between the WT and *trx y1y2*, since *trx y1y2* was reported to be sensitive to high light (Laugier et al. 2013).

In *trx x trx y1y2*, photoreduction of FBPase and SBPase was slightly delayed during photosynthesis induction (Fig. 3). Since the in vitro analysis showed that Trx *x* and Trx *y*, unlike Trx *f*, could not efficiently reduce FBPase and SBPase (Yoshida et al. 2015), the redox state of these enzymes may have been indirectly affected in *trx x trx y1y2*. NTRC also could not directly reduce FBPase and SBPase in vitro (Yoshida and Hisabori 2016), but photoreduction of these enzymes is severely suppressed in *ntrc* as an indirect effect of an imbalance in the chloroplast redox network (Perez-Ruiz et al. 2017). 2-Cys Prx is mainly reduced not only by NTRC but also by Trxs. In *ntrc*, Trxs would preferentially reduce the abundant 2-Cys Prx compared to other Trx target proteins. Accordingly, the redox imbalance in *ntrc* is partially alleviated by changing the electron flow by blocking electron transport from Trxs to 2-Cys Prx or from Fd to pathways other than Trxs. Lack of 2-Cys Prx or PGR5 partially restored photoreduction of FBPase and alleviated the growth defect observed in *ntrc* (Perez-Ruiz et al. 2017; Okegawa et al. 2022). Notably, 2-Cys Prx is also required for the recently discovered oxidative regulation mechanism of thiol enzymes (Vaseghi et al. 2018; Yoshida et al. 2018; Ojeda et al. 2018a). During the transition from light to dark conditions, Trx-like proteins oxidize and inactivate light dependently reduced Trx target proteins, whose reducing equivalents are transferred to 2-Cys Prx. Lack of Trx-like proteins also restored growth defects of *ntrc* (Yokochi et al. 2021).

Thus, 2-Cys Prx is important for maintaining the balance in the chloroplast redox network. Although Trx *x* is known to be the most efficient reductant of 2-Cys Prx in vitro (Collin et al. 2003), in vivo, no difference in its photoreduction was observed between the WT and *trx x* or *trx x trx y1y2* at least under the examined conditions (Fig. 6). In vivo, NTRC is the major reducing equivalent donor of 2-Cys Prx (Pulido et al. 2010), as the mutant phenotypes of *trx x* and *trx y* are only observed in the *ntrc* background under normal growth conditions (Ojeda et al. 2017; Jurado-Flores et al. 2020). Detecting the contribution of Trx *x* or Trx *y* to 2-Cys Prx reduction in vivo is challenging. However, differences that were not detectable by immunoblot analysis could possibly have a significant impact on plant growth. In fact, there was no difference in the redox state of 2-Cys Prx between *ntrc* and *trx x ntrc*, but a distinct difference in plant growth was observed (Ojeda et al. 2018b). Alternatively, real-time detection of the redox state of 2-Cys Prx with high temporal resolution using biosensors (Lampl et al. 2022) might reveal differences between the WT and mutants.

In addition to 2-Cys Prx, Prx Q also functions as a Trx oxidase (Telman et al. 2020). In the Trx oxidase assay, Trx *m*1 and Trx *m*4 oxidized the Trx target proteins and reduced Prx Q more efficiently than Trx *x*. However, Trx *x* and Trx *y* were found to reduce Prx Q more efficiently than Trx *f* and Trx *m* by measuring the Prx activity in vitro (Collin et al. 2004). Similar to the relationship between NTRC and 2-Cys Prx, if loss of Trx *x* and Trx *y* causes a disturbance in the redox balance of Prx Q, resulting in PSI acceptor-side limitation and growth defects, the mutant phenotype of *trx x* and *trx x trx y1y2* may be alleviated by loss of *prx q*.

Trx *x* and Trx *y* were originally known to play a photoprotective role (Collin et al. 2003, 2004). If oxidative stress is responsible for the mutant phenotype of *trx x* and *trx x trx y1y2*, mutants deficient in 2-Cys Prx or Prx Q may show a phenotype similar to that of *trx x* and *trx x trx y1y2* mutants. However, neither plant growth nor photosynthetic parameters were affected in *prx a* and *prx q* under fluctuating light conditions (Fig. 6; Supplemental Fig. S4). Alternatively, Trx *x* and Trx *y* may function in different phases under fluctuating light conditions. Trx *y* may not only serve as an assistant to Trx *x* but also function under conditions that allow it to be more efficient than Trx *x*. In addition, Prx IIE is also localized in chloroplasts (Dietz 2011). Deletions in each *Prx* gene may be complemented by other Prxs. Analysis of the *prx a prx q* double mutants might reveal the importance of the redox regulation of Prx by Trx *x* and Trx *y* under fluctuating light.

A critical question is why the acceptor side of PSI is limited in *trx x* and *trx x trx y1y2*, especially under fluctuating light. Light fluctuation is a potent stress to PSI, and PSI-CET is required to protect PSI from this stress (Supplemental Fig. S5A; Suorsa et al. 2012; Yamamoto and Shikanai 2019). High Y(NA) is caused by a shortage of electron acceptors from PSI or a defect in photosynthetic control. The *pgr5* mutant exhibits a high Y(NA) phenotype due to both defects, resulting in PSI photoinhibition (Yamamoto and Shikanai 2019). Slowing down the electron transport to PSI by the *pgr1* mutation alleviates PSI photoinhibition in *pgr5* under fluctuating light. Similarly, the high Y(NA) phenotype of *trx x trx y1y2* was mitigated by the *pgr1* mutation (Supplemental Fig. S3). In *pgr5*, however, PSI acceptor-side limitation is mainly observed at higher light intensities, in contrast to *trx x* and *trx x trx y1y2*, where a higher level of Y(NA) was induced at lower light intensities (Fig. 2). Furthermore, we previously reported that the PGR5-dependent pathway is regulated by Trx *m* but not by Trx *x* and Trx *y* (Okegawa and Motohashi 2020). In contrast, the NDH complex modulates the PSI acceptor-side redox state in the low-light phase of fluctuating light (Zhou et al. 2023). The NDH complex has been suggested to be redox regulated (Courteille et al. 2013; Nikkanen et al. 2018), although its detailed molecular mechanism remains unknown. NDH activity was not inhibited in *trx x* or *trx x trx y1y2* (Fig. 7), indicating that Trx *x* and Trx *y* are not essential for activation of NDH-dependent PSI-CET. However, the method used here to measure NDH activity is not quantitative; therefore, the possibility that Trxs are involved in the fine-tuning of NDH activity cannot be excluded.

So, then, how are Trx *x* and Trx *y* involved in preventing the redox imbalance of the PSI acceptor side? Trx *x* and Trx *y* accept electrons from FTR more efficiently (Yoshida and Hisabori 2017) and showed higher insulin reductase activity than other Trxs (Okegawa and Motohashi 2020). Considering their biochemical specificity, Trx *x* and Trx *y* may act as electron sinks during the sudden transition from low to high light. Furthermore, Trx *x* and Trx *y* are required to maintain the oxidized states of the PSI acceptor side under constant low-light conditions (Fig. 2). This function is seemingly unnecessary under constant light conditions; however, maintaining the redox balance on the PSI acceptor side in preparation for sudden changes in light intensity would be important for plant adaptability. Although we revealed the physiological importance of Trx *x* and Trx *y* in the acceptor-side regulation of PSI under fluctuating light conditions, the involved molecular mechanisms are still unknown. Further analysis is required to understand the photoprotective mechanisms mediated by Trx *x* and Trx *y*.

## Materials and methods

### Plant materials and growth conditions

*Arabidopsis* (*A. thaliana*) ecotype Col-0 was used as the WT. The T-DNA insertion line GK_179A03 (*trx x*) was obtained from the Nottingham Arabidopsis Stock Center (NASC, UK); SALK_103154 (*trx y1*), SALK_028065 (*trx y2*) (Laugier et al. 2013), GK_295C05 (*prx a*) (Perez-Ruiz et al. 2017), and SAIL_742_G10 (*prx q*) (Horn et al. 2020) were obtained from the Salk Institute Genomic Analysis Laboratory (SIGnAL, CA, USA). The *trx f1f2*, *pgr1*, *crr2*, and *pgr5* mutants have been previously described (Munekage et al. 2001, 2002; Hashimoto et al. 2003; Okegawa et al. 2020). The presence of mutations and T-DNA insertions was confirmed by PCR and sequence analysis (for primers, see Supplemental Table S1).

Plants were grown in soil or in Petri dishes containing MS medium with 1.0% (w/v) agar and 1% (w/v) sucrose and grown in growth chambers (50 to 60 *µ*mol photons m^−2^ s^−1^, 23 °C). For the growth of plants under fluctuating light, a timer was used to expose the plants to a cycle of 5 min of low light (30 *µ*mol photons m^−2^ s^−1^) and 1 min of high light (500 *µ*mol photons m^−2^ s^−1^).

### Isolation of chloroplasts and extraction of total leaf proteins

Leaves from 4- to 5-wk-old plants were homogenized with a Physcotron NS-52K homogenizer (Microtec, Japan) in 20 mm Tricine-NaOH, pH 8.4, containing 400 mm sorbitol, 5 mm MgCl_2_, 5 mm MnCl_2_, 2 mm EDTA, 10 mm NaHCO_3_, 0.5% (w/v) bovine serum albumin, and 5 mm ascorbate. After centrifugation at 2,000 × *g* for 5 min at 4 °C, the chloroplast pellet was gently resuspended in 50 mm HEPES-KOH, pH 7.6, containing 400 mm sorbitol, 5 mm MgCl_2_, and 2.5 mm EDTA and again centrifuged using the same settings. Isolated chloroplasts were suspended in 25 mm HEPES-KOH, pH 7.6, containing 3 mm MgCl_2_. The insoluble thylakoid membrane fraction was separated from the stromal fraction by centrifugation at 10,000 × *g* for 3 min at 4 °C. To extract total leaf proteins, seedlings grown under fluctuating light for 19 d (30-mg fresh weight) were frozen in liquid nitrogen and ground by tungsten beads (5-mm diameter). Total leaf protein was extracted in 62.5 mm Tris-HCl, pH 6.8, containing 2% (w/v) SDS, 10% (w/v) glycerol, 0.005% (w/v) bromophenol blue, and 50 mm dithiothreitol.

### SDS–PAGE and immunoblot analysis

Protein samples were separated by 12.5% or 15% SDS–PAGE using the conventional Laemmli (Tris-glycine) system (Laemmli 1970) or by 16.5% SDS–PAGE using a Tris-tricine buffer system (for PGR5 detection) (Schagger and von Jagow 1987) and transferred onto PVDF membranes. Specific antibodies against Trx isoforms, NTRC, FBPase, SBPase, RbcS, 2-Cys Prx AB, PetC, ATPC1, and PGR5 were described previously (Okegawa and Motohashi 2020; Murai et al. 2021). NdhL (a subunit of the NDH complex) antibody was a kind gift from Dr. Toshiharu Shikanai (Kyoto University). For other antibodies, commercially available polyclonal antibodies were used (Agrisera, Sweden). Immunoblot signals were visualized using the Chemi-Lumi One Super (Nacalai, Japan). The chemiluminescence signal was detected using a ChemiDoc XRS+ imaging system (Bio-Rad, USA).

### In vivo measurements of chlorophyll fluorescence and P700 absorption changes

Chlorophyll fluorescence and P700 absorption changes in the PSI reaction center were measured simultaneously using a portable chlorophyll fluorometer (DUAL-PAM-100 [MODULAR version] analyzer equipped with a P700 dual-wavelength emitter at 830 and 870 nm; Walz, Germany). The plants were kept in the dark for 30 min before each measurement, and detached leaves were used for the analysis. Minimum fluorescence (*F_o_*) was obtained using a weak measuring light (red light, 620 nm, 0.05 to 0.1 *µ*mol photons m^−2^ s^−1^). A saturating pulse (SP) (300 ms, 10,000 *µ*mol photons m^−2^ s^−1^) was applied to determine the maximum fluorescence in the dark-adapted state (*F_m_*) and during AL illumination (*F_m_*ʹ). The steady-state fluorescence level (*F_s_*) was recorded during AL illumination. *F_v_*/*F_m_*, Y(II), and NPQ were calculated as (*F_m_* − *F_o_*)/*F*_m_, (*F_m_*ʹ − *F_s_*)/*F_m_*ʹ, and (*F_m_* − *F_m_*ʹ)/*F_m_*ʹ, respectively (Genty et al. 1989). The *q_L_* was determined as described previously (Kramer et al. 2004).

The redox change of P700 was assessed by monitoring the absorbance changes to transmitted light at 830 and 875 nm. *P_m_* was determined by applying a SP in the presence of far-red light (720 nm). The maximal level of oxidized P700 during AL illumination (*P_m_*ʹ) was determined by a SP application. The P700 signal (*P*) was recorded immediately before a SP. Y(I) was calculated as (*P_m_*ʹ − *P*)/*P_m_*. Y(NA) was calculated as (*P_m_* − *P_m_*ʹ)/*P_m_*. Y(ND) was calculated as *P*/*P_m_*. Three complementary quantum yields were defined as follows: Y(I) + Y(NA) + Y(ND) = 1 (Klughammer and Schreiber 1994).

To analyze the effect of fluctuating light on photosynthesis, light treatment of leaves was performed using a DUAL-PAM-100 as described previously (Yamamoto et al. 2016). Briefly, leaves were illuminated with 5 min of low light (54 *µ*mol photons m^−2^ s^−1^) and 1 min of high light (1,455 *µ*mol photons m^−2^ s^−1^) for 3 cycles by changing the intensity of AL. Leaf discs (12 mm in diameter) taken from 4-wk-old plants grown under long-day conditions were used for measurements. After measuring the initial *F_v_*/*F_m_* and *P_m_*, the leaf discs were subjected to fluctuating light treatment and their photosynthetic parameters were monitored at that time. After the fluctuating light treatment, the leaf discs were sandwiched between wet tissue paper and incubated in the dark for 30 min, and then *F_v_*/*F_m_* and *P_m_* were measured. The degree of PSI and PSII photoinhibition is indicated as a percentage of the value of *F_v_*/*F_m_* and *P_m_*, respectively, after the fluctuating light treatment relative to the value before.

NDH activity was monitored using a Mini-PAM II (pulse-amplitude modulation) portable chlorophyll fluorometer (Walz, Germany) as described previously (Shikanai et al. 1998).

### In vivo photoreduction of thiol enzymes

Photoreduction of thiol enzymes (ATP synthase subunit CF_1_-γ, FBPase, and SBPase) in seedlings was determined using the free thiol-specific–modifying reagent 4-acetamido-4ʹ-maleimidylstilbene-2,2ʹ-disulfonic acid (Thermo Fisher Scientific, USA) as described previously (Okegawa and Motohashi 2020). Seedlings were dark adapted for 8 h and exposed to light (150 *μ*mol photons m^−2^ s^−1^) for up to 30 min. In vivo redox state of 2-Cys Prx was assessed during the transition from low light (30 *µ*mol photons m^−2^ s^−1^) to high light (500 *µ*mol photons m^−2^ s^−1^) as described previously (Vaseghi et al. 2018). Reduced thiols were blocked by adding 100 mm*N*-ethylmaleimide in extraction buffer. Samples were collected at the indicated time points and analyzed by immunoblot analysis. The reduction level of proteins was quantified using Multi Gauge 3.1 software (Fujifilm, Japan) and presented as the ratio between reduced protein and total protein.

### Analysis of chlorophyll content

Leaves (30-mg fresh weight) were immediately powdered by grinding in liquid nitrogen. Chlorophyll was extracted in 80% acetone (v/v). The chlorophyll content was determined by spectrophotometry as described (Porra et al. 1989).

### Statistical analysis

Calculations were performed on more than 3 independent biological replicates (see figure legends). Tukey's multiple comparison test was used to determine significant differences among the materials tested (*P* < 0.05).

### Accession numbers

Sequence data from this article can be found in the Arabidopsis Genome Initiative or GenBank/EMBL databases under the following accession numbers: Trx *x* (At1g50320), Trx *y*1 (At1g76760), Trx *y*2 (At1g43560), Trx *f*1 (At3g02730), Trx *f*2 (At5g16400), PGR1 (At4g03280), 2-Cys Prx A (At3g11630), Prx Q (At3g26060), CRR2 (At3g46790), and PGR5 (At2g05620).

## Supplementary Material

kiad466_Supplementary_Data

## Supplemental data

**Supplemental Figure S1.** Identification of the *trx x* T-DNA insertion mutant.

**Supplemental Figure S2.** Chlorophyll fluorescence and P700 parameters during steady-state photosynthesis in WT Col-0, *trx x*, *trx y1y2*, and *trx x trx y1y2* plants.

**Supplemental Figure S3.** Impact of decreased electron input to PSI by the *pgr1* mutation on the photosynthetic parameters of *trx x trx y1y2* plants under fluctuating light.

**Supplemental Figure S4.** Characterization of the *prx a* and *prx q* mutants under fluctuating light.

**Supplemental Figure S5.** Visible phenotypes of WT Col-0, *trx x trx y1y2*, *crr2*, and *pgr5* plants grown under fluctuating light.

**Supplemental Table S1.** Primers used in this study.

## Dive Curated Terms

The following phenotypic, genotypic, and functional terms are of significance to the work described in this paper:

ATPC1 Gramene: AT4G04640
ATPC1 Araport: AT4G04640

CRR2 Gramene: AT3G46790
CRR2 Araport: AT3G46790

ATP CHEBI: CHEBI:15422

chlorophyll CHEBI: CHEBI:28966

pgr5 Gramene: AT2G05620
pgr5 Araport: AT2G05620
