# Supplementary material for: x- and y-type thioredoxins maintain redox homeostasis on photosystem I acceptor side under fluctuating light
Source: Plant Physiol. 2023 Aug 22;193(4):2498–512. doi: 10.1093/plphys/kiad466 (PMC10663110; doi:10.1093/plphys/kiad466)
Supplement: kiad466_Supplementary_Data [file kiad466_supplementary_data.pdf]

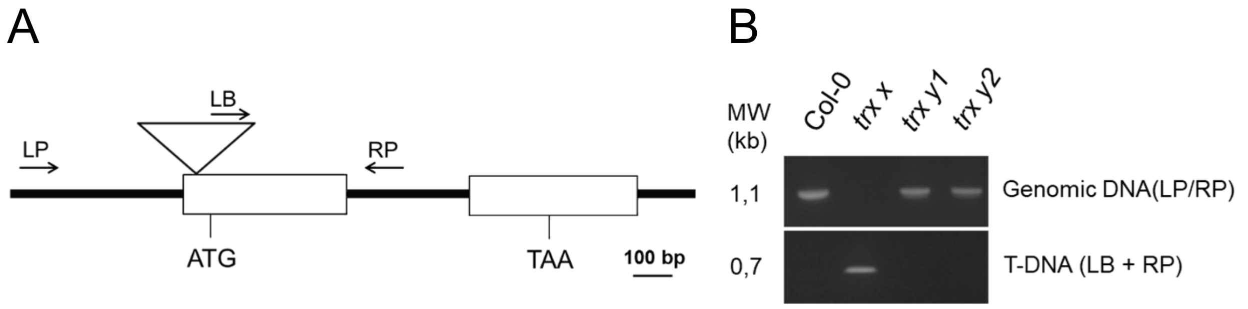

Supplemental Figure S1. Identification of the *trx x* T-DNA insertion mutant.
A, Schematic diagram of the structure of the *Trx x* gene. The T-DNA insertion site is indicated in the diagram by the upside-down triangle and its nucleotide position is 30 bp upstream of ATG. White boxes indicate coding regions. Arrowheads indicate the positions of primers used for genotyping. B, Genotyping of the *trx x* (GK_179A03) mutant. The presence of the T-DNA insertion was tested by PCR using genomic DNA isolated from the wild type (WT) Col-0, *trx x*, *trx y1*, and *trx y2* plants. The WT and mutant genomes are represented by bands of approximately 1.1 and 0.7 kb, respectively. LP, left primer; RP, right primer; LB, left border primer for T-DNA.

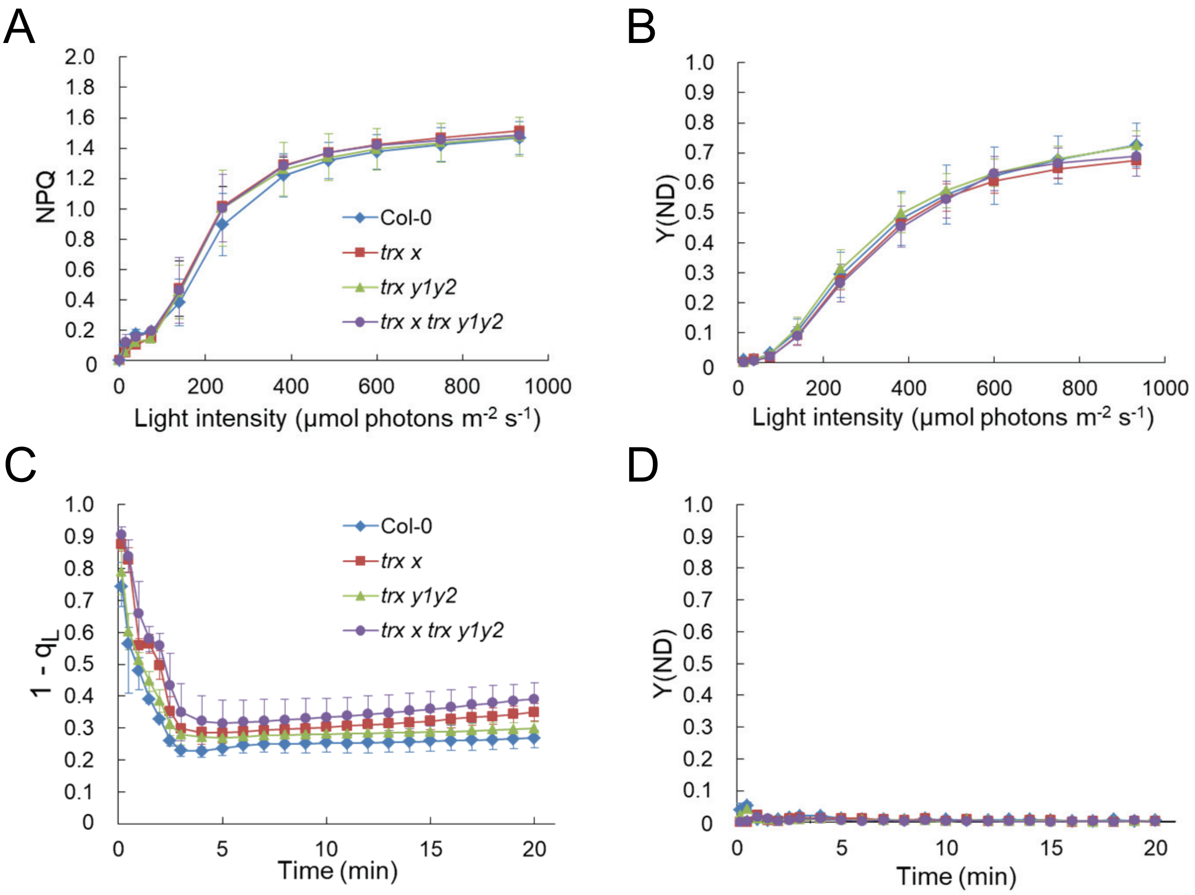

Supplemental Figure S2. Chlorophyll fluorescence and P700 parameters during steady-state photosynthesis in WT Col-0, *trx x*, *trx y1y2*, and *trx x trx y1y2* plants.

A and B, The light intensity dependence of non-photochemical quenching (NPQ) of chlorophyll fluorescence (A) and donor-side limitation of PSI [Y(ND)] (B). Each value is the mean ± standard deviation (SD) of three independent plants. C and D, Reduction level of the PQ pool (1 – q_L) (C) and Y(ND) (D) measured upon illumination at 64 µmol photons m^{-2} s^{-1} for 20 min after acclimation to darkness for 30 min. Each data point represents the mean ± SD (*n* = 6–8 independent plants).

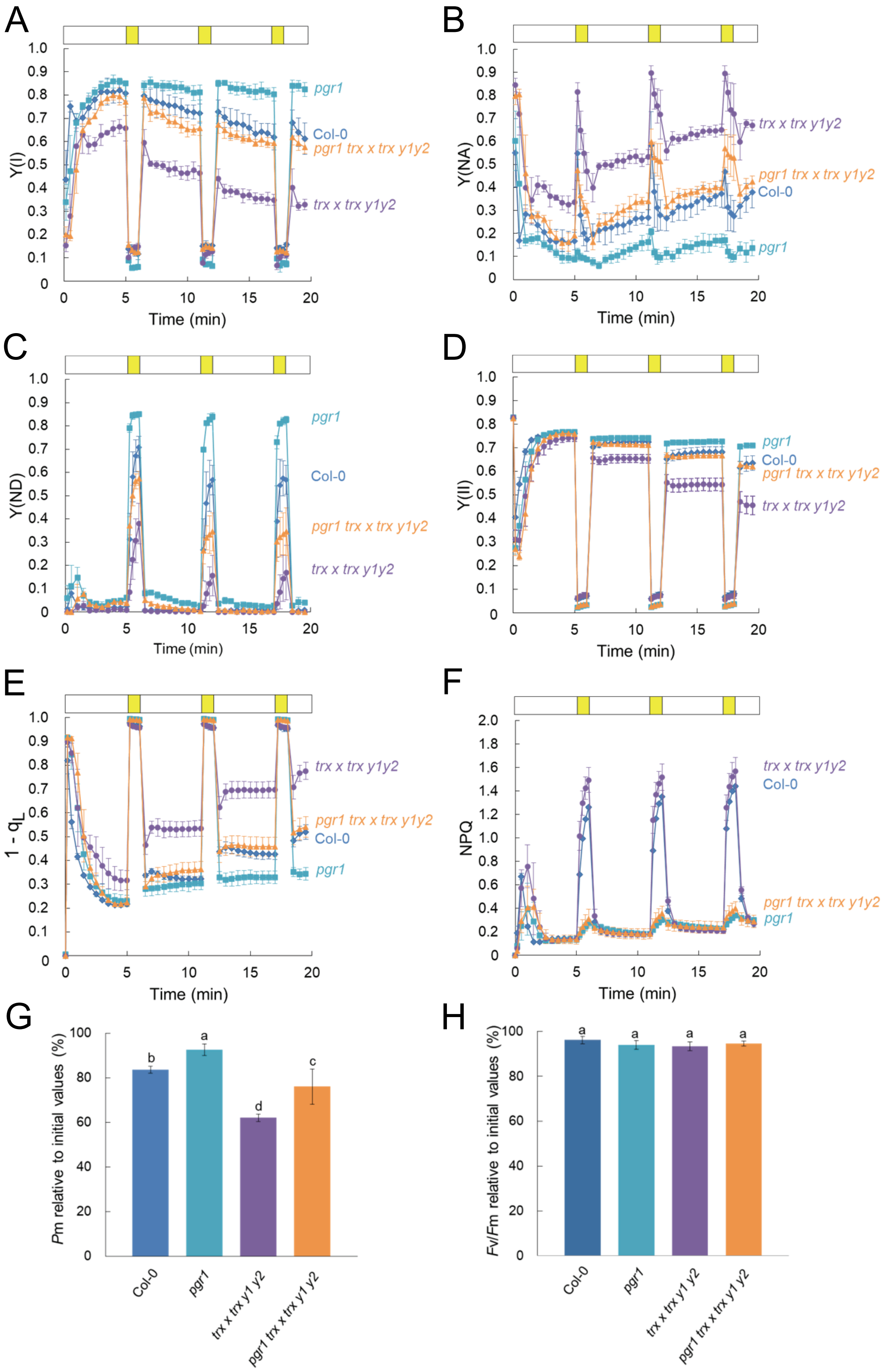
A
B
C
D
E
F
G
H
Y(I)
Y(NA)
Y(ND)
Y(II)
1 - q_L
NPQ
Time (min)
pgr1
Col-0
pgr1 trx x trx y1y2
trx x trx y1y2
Pm relative to initial values (%)
Fv/Fm relative to initial values (%)
Col-0
pgr1
trx x trx y1 y2
pgr1 trx x trx y1 y2
a
b
c
d

Supplemental Figure S3. Impact of decreased electron input to PSI by the *pgr1* mutation on the photosynthetic parameters of *trx x trx y1y2* plants under fluctuating light.

Chlorophyll fluorescence and P700 parameters were measured under fluctuating light consisting of 5 min of low light (white bar, 54 μmol photons m^{-2} s^{-1}) and 1 min of high light (yellow bar, 1,455 μmol photons m^{-2} s^{-1}) in WT Col-0, *pgr1*, *trx x trx y1y2*, and *pgr1 trx x trx y1y2* plants grown under long-day conditions. A–F represent photochemical quantum yield of PSI [Y(I)], acceptor-side limitation of PSI [Y(NA)], Y(ND), effective quantum yield of PSII [Y(II)], 1 – q_L, and NPQ, respectively. Each data point represents the mean ± SD (n = 3 independent plants). G and H, PSI and PSII photoinhibition. The values of maximum oxidizable P700 (Pm) and maximum quantum yield of PSII (F_v/F_m) were compared before and after the fluctuating light treatment. Each value is the mean ± SD (n = 3–6 independent plants). Different letters indicate statistical differences confirmed by the Tukey-Kramer test (P < 0.05).

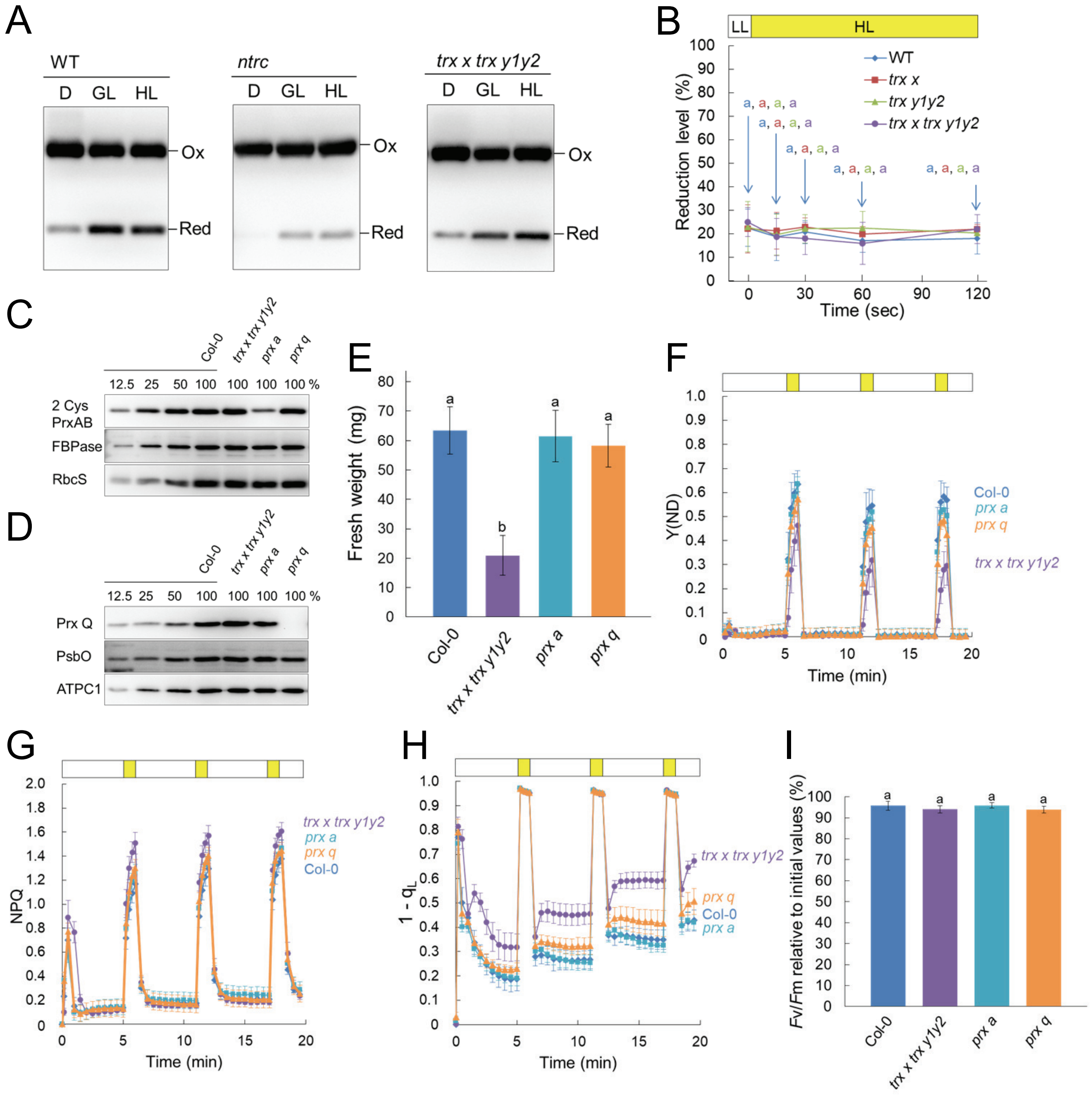
A
WT
D GL HL
Ox
Red
ntrc
D GL HL
Ox
Red
trx x trx y1y2
D GL HL
Ox
Red
B
LL
HL
WT
trx x
trx y1y2
trx x trx y1y2
Reduction level (%)
Time (sec)
a, a, a, a
C
Col-0
trx x trx y1y2
prx a
prx q
12.5 25 50 100 100 100 100 %
2 Cys PrxAB
FBPase
RbcS
D
Col-0
trx x trx y1y2
prx a
prx q
12.5 25 50 100 100 100 100 %
Prx Q
PsbO
ATPC1
E
Fresh weight (mg)
Col-0
trx x trx y1y2
prx a
prx q
F
Y(ND)
Time (min)
Col-0
prx a
prx q
trx x trx y1y2
G
NPQ
Time (min)
trx x trx y1y2
prx a
prx q
Col-0
H
1 - qL
Time (min)
trx x trx y1y2
prx q
Col-0
prx a
I
Fv/Fm relative to initial values (%)
Col-0
trx x trx y1y2
prx a
prx q

Supplemental Figure S4. Characterization of the *prx a* and *prx q* mutants under fluctuating light. A, Photo-reduction of 2-cysteine peroxiredoxin (2-Cys Prx) under different light intensities in WT Col-0, *ntrc*, *trx x trx y1y2*. After 8 h in dark, seedlings were subjected to illumination of 50 (GL) or 500 (HL) μmol photons m^{-2} s^{-1} for 1 h. Samples were collected and modified with N-ethylmaleimide. The redox state of 2-Cys Prx was detected by immunoblot analysis. B, The reduction levels of 2-Cys Prx (Figure 6A) are indicated as a percentage of the total protein that was reduced. Seedlings were exposed to LL (30 μmol photons m^{-2} s^{-1}) for 30 min, then to HL (500 μmol photons m^{-2} s^{-1}) for 120 sec and collected at the indicated time points. Each value represents the mean ± SD (n = 5–7 independent plants). Means with the same letters are not significantly different between genotypes according to Tukey's test, P < 0.05. C and D, Accumulation of 2-Cys Prx and Prx Q in WT Col-0, *trx x trx y1y2*, *prx a* and *prx q* plants. Chloroplasts were fractionated into stromal fractions and thylakoid membranes. Ten micrograms of stromal protein (C) or thylakoid membrane proteins corresponding to 1.0 μg chlorophyll (D) were loaded per lane, as well as a dilution series of WT proteins. Antibodies used are indicated on the left. E, Fresh weight of seedlings grown under fluctuating light (Figure 6B). Each value is shown as the mean ± SD (n = 10 independent plants). Different letters indicate statistical differences confirmed by the Tukey-Kramer test (P < 0.05). F–H, Analysis of chlorophyll fluorescence and P700 parameters under fluctuating light in WT Col-0, *trx x trx y1y2*, *prx a*, and *prx q* plants. Four-week-old plants grown under long-day conditions were exposed to cycles of low light (white bar, 54 μmol photons m^{-2} s^{-1}) and high light (yellow bar, 1,455 μmol photons m^{-2} s^{-1}). F–H represent Y(ND), NPQ, and $1 - q_L$, respectively. Each data point represents the mean ± SD (n = 5–10 independent plants). I, PSII photoinhibition. Each value is the mean ± SD (n = 6–8 independent plants). Means with the same letters are not significantly different between genotypes according to Tukey's test, P < 0.05. Ox, oxidized; Red, reduced; D, dark; GL, growth light, HL, high light; LL, low light.

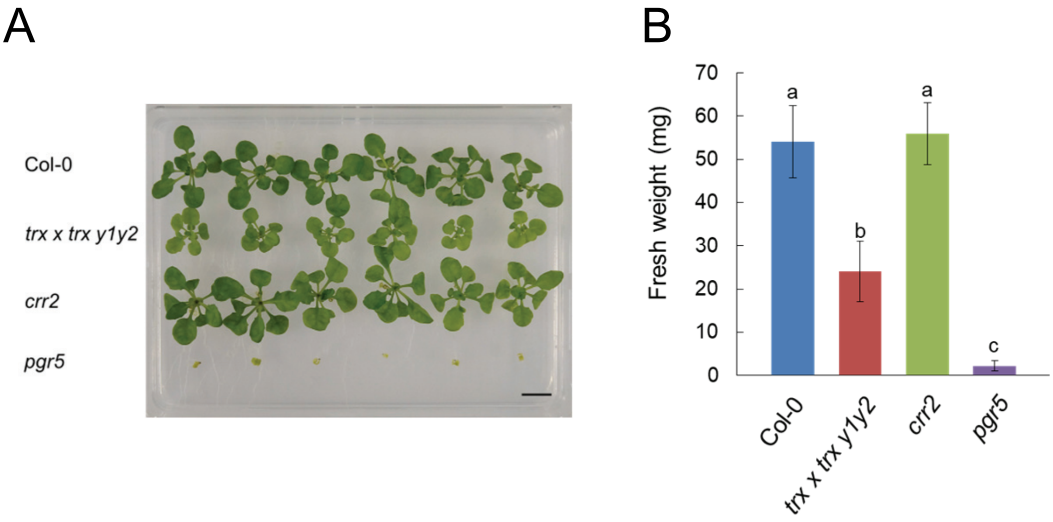

Supplemental Figure S5. Visible phenotypes of WT Col-0, *trx x trx y1y2*, *crr2*, and *pgr5* plants grown under fluctuating light.

A, Seedlings were grown for 19 days in growth chambers under fluctuating light cycles of 5 min of low light (30 μmol photons m^{-2} s^{-1}) and 1 min of high light (500 μmol photons m^{-2} s^{-1}). Scale bars, 10 mm. B, Fresh weight of seedlings grown for 19 days under fluctuating light. Each value is shown as the mean ± SD (n = 10–15 independent plants). Different letters indicate statistical differences confirmed by the Tukey-Kramer test ($P < 0.05$).

Supplemental Table S1. Primers used in this study.

The following primers used for genotyping:

Trx *x*-LP	5'-TTTAAACAGGAATGTTGATGCTATG-3'
Trx *x*-RP	5'-CAAAAGGAAGCTCAACTCTAAACAG-3'
Trx *y*1-LP	5'-GGTTCCCCATCTTTGAATAGG-3'
Trx *y*1-RP	5'-AACATGTGGGCCTAGAACATG-3'
Trx *y*2-LP	5'-CGATGGATTAGCGAACTATGC-3'
Trx *y*2-RP	5'-GAGCAAACAATCAACAATGGC-3'
LB (GK)	5'-ATATTGACCATCATACTCATTGC-3'
LBb1.3 (SALK)	5'-ATTTTGCCGATTTCGGAAC-3'
PGR1-F	5'-TTAGCCCTCCTTGAAACACTG-3'
PGR1-R	5'-ACCCATGGAACAAAAAGAACC-3'
